# Mesenchymal Stem Cell-Derived Extracellular Vesicles Therapy for Pulmonary Hypertension: A Comprehensive Review of Preclinical Studies

**DOI:** 10.1155/2022/5451947

**Published:** 2022-11-04

**Authors:** Ji-Hong Xu, Jia-Ping Liang, Chu-Jun Zhu, Yu-Jun Lian

**Affiliations:** ^1^Department of Anesthesiology, Shenzhen University General Hospital, Shenzhen, China; ^2^Shenzhen University, Shenzhen, China

## Abstract

Pulmonary hypertension (PH) is a type of clinical pathophysiological syndrome characterized by a progressive increase in pulmonary vascular resistance and subsequent progressive failure of the right heart function, and is a common complication of many diseases. Mesenchymal stem cells (MSCs) autonomously home to sites damaged by disease, repair damaged tissues, and participate in the regulation of systemic inflammation and immune responses, which have good clinical application prospects. Extracellular vesicles (EVs), such as exosomes and microvesicles, participate in various biological activities by regulating intercellular communication. Exosomes secreted into the extracellular environment also affect the host immune system. MSC-derived extracellular vesicles (MSC-EVs), as a mediator in the paracrine processes of MSCs, carry biologically active substances such as proteins, lipids, mRNA, and micro-RNA. MSC-EVs therapies, safer than cell-based treatments, have been shown to be effective in modulating macrophages to support anti-inflammatory phenotypes, which are strongly related to histological and functional benefits in preclinical models of pulmonary hypertension. The main effects of active substances and their potential medical value have attracted wide attention from researchers. This article reviews the role and relevant mechanisms of MSC-EVs in the treatment of pulmonary hypertension in recent studies and provides a basis for their future clinical applications.

## 1. Introduction

Pulmonary hypertension (PH) is a chronic severe cardiopulmonary disease caused by pulmonary vascular remodeling, characterized by progressive narrowing of blood vessels and elevated mean pulmonary arterial pressure, ultimately leading to heart failure and death. The main pathological change of PH is pulmonary vascular remodeling, which includes antiapoptosis and proliferation of pulmonary artery endothelial and smooth muscle cells, muscularization of the distal pulmonary artery, production and deposition of proinflammatory cytokines and chemotactic cytokines, and accumulation and infiltration of leukocytes around the pulmonary blood vessels [1–4]. PH is also a life-threatening condition of bronchopulmonary dysplasia (BPD) in premature infants [5]. Statistics show that more than 40,000 people worldwide suffer from PH each year and the fatality rate is as high as 15% [6]. Even if patients with PH undergo treatment, the 3-year survival rate is only 55%–65%. The current drugs on the market are not very effective in treating pulmonary hypertension. Most patients have a poor prognosis and little improvement in their quality of life. Furthermore, the drugs are expensive and often accompanied by obvious side effects [7]. Therefore, a new drug that cures or controls the development of PH is urgently needed in the clinic. Extracellular vesicles (EVs) are released by various types of cells, mediating intercellular communication, transmitting specific information from their origin cells to target cells, and thus, participating in physiological and pathophysiological processes. Due to these properties, EVs from specific types of cells can be used as new agents to treat various diseases, including antitumor treatment, immunomodulation, regenerative therapy, pathogen vaccination, and drug delivery. Translating EVs into clinical treatments requires the classification of EV-based therapies according to existing regulatory frameworks [8]. Mesenchymal stromal cells (MSCs) and EVs have been evaluated in patients with PH through a series of Phase I/II trials. The efficacy and safety of adipose-derived mesenchymal stem cells for PH are being assessed [9]. Preclinical studies have shown that MSC-EVs improve pulmonary hypertension [10, 11], and the emergence of exosomes has brought hope down to patients with pulmonary hypertension.

## 2. Biological Characteristics of Extracellular Vesicles

Extracellular vesicles (EVs) are membrane structures that are naturally released from cells. They are delimited by lipid bilayers and cannot be replicated, i.e., do not contain functional nuclei. EVs include exosomes (Exos), microvesicles (MV), microparticles (MP), exosomes, tumor bodies, and apoptosomes [12]. The term “exosome” is most commonly used to specify any type of EVs [13]. The International Society for Extracellular Vesicles (ISEV) supports “extracellular vesicle” (EV) as a generic term for particles. Because of overlapping size ranges and lack of specific markers, current extracellular vesicle preparations, including exosome preparations are heterogenous with undefined biogenesis origins and undetermined purity. The separation of EVs from other non-EV components of the matrix (conditioned media, biological fluids, and tissues) and between different types of EVs (commonly known as purification or isolation) is achieved through the different techniques available. Characterization of EVs through multiple complementary techniques is important to evaluate the effectiveness of isolation techniques and to determine the similarity of biomarkers or functions. To characterize a single EV, as a general rule, at least two techniques should be used [12].

Extracellular vesicles, especially exosomes, have been well documented as carriers of biomarkers in the extracellular space [14]. It was first discovered in 1983 that mature reticulocytes in sheep released a membrane vesicle that was originally thought to excrete excess transferrin receptors, known as exosomes [15]. Exosomes are single membrane vesicles approximately 30–100 nm in size that are widely distributed in saliva, plasma, and milk [16–18]. Exosomes have a bimolecular phospholipid structure. The surface is rich in lipids such as cholesterol, sphingomyelin, and ceramide. It contains protein, mRNA, and micro-RNA and plays a vital role in the cellular microenvironment [19, 20]. When EVs circulate, they are absorbed by adjacent or distant cells through membrane fusion, endocytosis, and ligand-receptor binding, directly affecting gene expression and cellular phenotype of recipient cells, which are considered to be major players in many biological processes under normal and pathological backgrounds [21, 22].

### 2.1. Origin of EVs/Exosomes

Exosomes originate from late endosomes of the endocytic system. The formation process consists of two parts. First, exosomal membrane buds form inward vesicles by reverse budding, and part of the cytoplasm becomes multivesicular cells. Then, the multicarrier endosomes fuse with the cell membrane, and the internal vesicle structure is released outside the cell to form exosomes [23–25]. Exosomes express CD55 and CD59 on the surface, thus, avoiding activation of opsonins, complement, and coagulation factors [26]. They have phagocytosis, are able to pass relatively freely through the vascular wall and extracellular matrix and are widely and stably distributed in body fluids. Exosomes eventually undergo membrane fusion with surrounding cells, enabling the circulation of biofilms.

### 2.2. Structure of EVs

Glioblastoma tumor cells release microvesicles containing 41,860 kinds of proteins, more than 7,540 kinds of RNA, and 1,116 kinds of lipid molecules, as well as specific glycoconjugates and membrane-related advanced oligomeric protein complexes [27]. However, ISEV noted that the described and characterized proteins should be present in the EVs of the target as expected and have transmembrane functions (e.g., transmembrane proteins) and membrane binding capabilities (e.g., cytosolic proteins) [13].

EVs vary in shape, size, and even content in different biological states and stages of formation. Generally, the content of EVs from different sources varies.

Proteins include membrane transport proteins, fusion proteins (e.g., GTPases, Annexins, and Flotillin), transmembrane proteins (e.g., CD9, CD63, CD81, and CD82), heat shock proteins (e.g., HSP70 and HSP90), and proteins involved in vesicle biosynthesis (e.g., ALG-2, ALG-2-interacting protein X (Alix), tumor susceptibility gene 101 (TSG101), and lipid-related proteins) [28–32].

Exosomes contain some nucleic acids, such as noncoding RNA, mRNA, and siRNA. After these RNAs are absorbed by the target cells, they express proteins corresponding to the mRNAs. They also degrade target RNA or interfere with its translation to mediate the silencing of target genes, thereby influencing the physiological activity of cells in vivo. Exosomes with rich RNA content are mainly involved in the transmission of genetic information between tissues. Exosomes with low RNA content those from immune cells; mainly play a major role in antigen presentation and immune co-stimulation [31]. Studies have shown that exosomes from mast cells contain mature miRNAs that can be transferred to other cells and function in new cells [33]. Therefore, these nucleic acid molecules can also serve as biomarkers for tumor diagnosis and detection, enabling relevant targeted therapies, some of which have been identified as serum markers for cancer [34].

MVs also contain antigens, such as major histocompatibility complex (MHC) I and MHCII molecules, which activate T lymphocytes, natural killer cells, and dendritic cells, stimulating biological effects on immunity [35].

In addition to protein and nucleic acids, exosomes are also rich in lipids, such as cholesterol (mainly secreted by B lymphocytes), sphingolipids containing ceramide, and phosphoglycerides with long saturated fatty acyl chains [36]. Lipids in exosomes may be associated with lipid transport and membrane fusion in target cells where the membrane surface is rich in sugars such as mannose, polylactosamine, *α*-2,6 sialic acid, and complex N-linked sugar chains. These substances play an important role in protein sorting [37].

### 2.3. Mode-of-Action of EVs

EVs/exosomes have been used as a tool for directed communication between stem cells and parenchymal cells, enabling them to act as biomarkers [38]. Currently, exosomes are thought to transfer information between cells through four main mechanisms. (1) Exosomes recognize and fuse with target cells via their own membrane proteins [39]. (2) After the lipids in exosomes are transported to recipient cells, they promote apoptosis through the Notch signaling pathway [40]. (3) Transcription factor receptors from exosomes regulate transcription in target cells [41]. (4) Exosome-derived microRNAs bind to silencing complexes and play a role in post-transcriptional regulation of recipient cells [42]. Once EVs are absorbed by the recipient cell, lipids, proteins, mRNA, micro-RNA, and other components contained therein influence protein modification and localization by altering transcription and translation procedures, and regulating signaling cascades and important enzymatic reactions. Methods such as self-regulation affect the cellular phenotype and state of target cells, while the types of source and recipient cells and their physiological and pathological states determine which mechanism plays a major role [43].

However, it should be noted that increasing studies indicate that miRNAs are not present in functional supervisor states or concentrations and are rarely delivered to cells. Authors should clarify the facts and proceed with caution to avoid confusion [44]. Most exosomes derived from standard formulations do not contain many copies of miRNA molecules. Therefore, reassessment of current exosome-mediated miRNA communication mechanisms and associated chemometric analysis is necessary to study other extracellular vesicles and their associated RNAs [45].

### 2.4. Role of EVs in PH

Because EVs contain many molecules, such as miRNAs, mRNAs, and proteins, these intercellular communicators act as biomarkers of lung disease, providing critical information about the health and disease status of donor cells [46]. An earlier study published in 2008 showed that endothelial cell-derived CD105 microparticles increased in the pulmonary artery blood of patients with pulmonary arterial hypertension (PAH) [47]. Furthermore, another study reported elevated levels of endothelial PECAM and VE-cadherin in plasma samples of patients with PH compared to the control group, but no increase in E-selective proteins. An increase in endothelial cell-derived MPs is associated with an increase in mean pulmonary arterial pressure, pulmonary vascular resistance, and mean right arterial pressure [48]. Subsequent studies have shown that patients with PH have a higher number of CD62e + endothelial cell-derived MPs in their plasma [49].

Both proteins and RNAs in the EV content are thought to be critical in the pathogenesis of PH. It was found that translation-controlled tumor proteins were highly expressed in exosomes released by blood-grown endothelial cells with BMPR-2 mutations, which was associated with heritable PH [50].

## 3. EVs Derived from Stem Cells

### 3.1. Classification and Characteristics of Stem Cells

Stem cells are cells that self-renew and differentiate into cells with specific functions under certain signaling conditions [51]. Stem cells are not only progenitor cells but also candidate cells. There are a large number of various stem cell types. When tissues age or are damaged, resting stem cells receive signals, perform physiological functions, proliferate, differentiate, repair, and replenish aging and damaged tissues, thereby maintaining the normal tissue structure and physiological functions. The properties of stem cells in repair and regeneration have opened up new ideas for the treatment of many diseases and have become research hotspots. There are many types of stem cells and their functions are also different. At present, there are two main classification methods. (1) In accordance with their development process, they are divided into three types: embryonic stem cells, germ cells, and adult stem cells. (2) In accordance with their differentiation potential, they are divided into four types: totipotent stem cells, subpotent stem cells, pluripotent stem cells and unipotent stem cells [52–54].

Embryonic stem cells have unique miRNAs and proteins that promote the proliferation and differentiation of endogenous progenitor cells, and embryonic stem cells are promising sources of exosomes. Adult stem cells are slow-circulating cells that respond to specific environmental signals. They are widely distributed and sourced, and the material is relatively readily available. They can also be autologously transplanted. The above advantages make them the first choice for stem cell therapy. Mesenchymal stem cells are adult pluripotent cells with self-renewing and differentiated mesenchymal plasma lineages, namely osteoblasts, fat cells, and chondrocytes, and are widely used in the field of regenerative medicine [55, 56]. The cells have the characteristics of easy isolation, high proliferation, easy in vitro, amplification, and genetic stability [57].

### 3.2. Effect of EVs/Exosomes on Mesenchymal Stem Cell Therapy

In 2009, researchers discovered that mesenchymal stem cells exerted their therapeutic effects through extracellular vesicles. Specifically, they found that extracellular vesicles, called microvesicles, which were 80–1000 nm in size, might prevent acute tubular injury [58]. Subsequent studies showed that 50–200 nm extracellular vesicles (i.e., exosomes) were effective in preventing myocardial reperfusion injury. This is the first time that mesenchymal stem cell-derived exosomes (MSC-Exos) have been confirmed [59]. Additionally, the term MSC-Exos is often used to describe 50–200 nm EVs prepared from MSC-conditioned media, as these EVs collectively contain exosome-associated proteins including TSG101, Alix, and tetramer transmembrane proteins CD9, CD63, and CD81. They also carry a rich variety of RNAs. MSC-Exos are increasing recognized as effective mediators for increasing number of MSC-related therapies [60].

Theoretically, exosomes are paracrine effectors of mesenchymal stem cells, which have important activities of their origin cells in a range of different disease models, including differentiation into polylines, cytokine secretion, promotion of cell proliferation, and immunomodulation. MSC-Exos have functional characteristics similar to those of stem cells. The immune potency of MSCs resides in their secreted small extracellular vesicles (sEVs). These sEVs have a large number of proteins with potent immunomodulatory activity [61]. In in vitro and in vivo models, MSC-EVs suppressed proinflammatory processes and oxidative stress in humoral and cellular components of the immune system, creating a proregenerative environment [62]. They are immunocompetent nanoparticles that mediate immune activity by stimulating T cell proliferation and IFN-*γ* secretion, thereby inhibiting the production of anti-CD3 and anti-CD28 antibody [63]. Additionally, MSC-Exos freely cross the blood-brain barrier and manipulate a large number of physiological and pathological processes by influencing the survival, proliferation, migration, and gene expression of recipient cells [64]. The content of exosomes derived from MSCs has its own characteristics. MSCs and their environments transmit feedback information to each other through MSC-Exos. Thus, changes in the environment of MSCs alter the amount of exosomes they secrete, which affects and alters the tissue environment. Mesenchymal stem cells produce more exosomes than myoblasts, human acute monocyte leukemia cell lines, and human embryonic renal cell lines [65]. Therefore, MSCs are considered to have the strongest exosome secretory capacity. By passing information, such as proteins, genetic material, and lipids, between cells, exosomes are important players in cell-to-cell communication [66]. MSC-Exos and exosomes from other sources do not differ significantly in morphology, isolation, or storage. MSC-Exos express common surface markers of exosomes, such as CD9, CD63, and CD81, as well as some adhesion molecules of the MSCs membrane, such as CD29, CD44, and CD73, among others [67, 68]. They are a simple and fast approach to administration, which improves the safety of treatment and reduces storage and transportation problems as well as many of the risks related to cell transplantation. Therefore, exosomes from mesenchymal stem cells are attractive as potential therapeutic agents.

Extensive research has been conducted on the application of MSC-Exos in the field of lung diseases, mainly focusing on pneumonia, lung dysplasia, lung tissue damage, and their terminal states. Other than that, MSC-Exos has been applied to bronchopulmonary diseases such as pulmonary dysplasia and radiation damage. Bronchopulmonary dysplasia is more common in children with perinatal acute respiratory failure. Bone marrow mesenchymal stem cells significantly resist oxygen radical-induced bronchopulmonary dysplasia. This mechanism may be associated with upregulation of the antioxidant stanniocalcin-1 in MSC-Exos. They are also associated with antiapoptotic effects [69]. MSCs are considered to be promising drugs for the treatment of radiation-induced lung damage. MSC-Exos mitigates radiation damage. Their mechanisms may be related to immunomodulatory, antifibrotic, regenerative, and restorative functions of exosomes [70].

Small extracellular vesicles derived from the mesenchymal matrix/stem cells (MSCs-sEV) are rapidly being applied in the clinical practice phase. However, there is still controversy about the biology, function, and potency of MSCs-sEVs. To resolve these controversies, four societies (SOCRATES, ISEV, ISCT, and ISBT) have proposed standards for MSC-sEVs to facilitate data sharing and comparison, which will propel the field toward clinical applications [71].

## 4. EVs Derived from MSCs Treat PH

### 4.1. MSC-Derived EVs Attenuate Experimental PH

MSC-EVs carry genetic information and proteinaceous material to modulate interactions between MSCs and recipient cells. Recently, more and more researchers have begun to focus on the medical role of MSC-EVs in chronic respiratory diseases. MSC-EVs are derived from the umbilical cord, bone marrow, adipose tissue, and induced pluripotent stem cells. Alternative modes of administration are intratracheal and intravenous injection. Treatments include chronic obstructive pulmonary disease, asthma, pulmonary fibrosis, and PH [72].

In animal studies, injection of MSC-EVs before or after induction of PH reversed the increase of right ventricular (RV) systolic pressure and RV hypertrophy, as well as decreased peripheral pulmonary vascular muscularization [9, 73, 74] (Table 1). The MSC-MVs exhibited general morphological characteristics of MVs, rapid annexin V and CD29 markers under TEM, with sizes ranging from 40 to 300 nm. Intravenous MSC-MVs or MSCs significantly improved mean pulmonary arterial pressure and mean RV pressure in PH rats. In addition, intravenous administration of MSC-MVs or MSCs significantly reduced RV hypertrophy, the pulmonary arteriole area index, and the thickness index in rats with PH [75, 76] (Table 1). The MSC-MVs also relieved the inflammation score and collagen fiber volume fraction [76] (Table 1). MSC-EVs attenuated monocrotaline-induced pulmonary vascular remodeling in mice with PH, an effect mediated directly by EVs on the pulmonary vascular system or through differentiation of myeloid cells into endothelial progenitor cells [77] (Table 1), [82]. These studies have revealed that MSC-Exos treatment could significantly suppress pulmonary vascular remodeling and EndMT. In another experiment, EVs from bone marrow, lungs, and plasma inhibited apoptosis of pulmonary vascular endothelial cells [46].

In cell culture experiments, MSC-Exos significantly inhibited hypoxia-induced apoptosis of pulmonary artery endothelial cells and proliferation of pulmonary artery smooth muscle cells [9] (Table 1). These data support that MSC-EVs can be a new treatment for PH [74, 83].

The use of MSC-EV at lower doses and with longer dosing intervals than previously reported can also be effective in reversing SuHx pulmonary hypertension. Hypoxic stress did not enhance this effect. These findings provide a basis for the feasibility of MSC-EV as a long-term treatment for pulmonary hypertension [78] (Table 1).

Adipose mesenchymal stem cells-derived Exos (ASC-Exos) prevented PH. Experimental results of coculture of human pulmonary artery endothelial cells (HPAECs) and ASCs-Exos showed that ASCs-Exos promoted proliferation of HPAECs treated with monochloride pyrrole [79] (Table 1).

MSC-EVs therapy had the potential to reverse CDH-related changes, particularly by rapidly inhibiting ECM-remodeling enzymes (LOX and MMP-9). Besides that, MSC-EVs therapy enhanced the structural aspects of the pulmonary arterial extracellular matrix and mitigated pathological disorders such as increased medial wall thickness and stiffness [80] (Table 1).

Early and late MSC-derived small extracellular vesicles (MEx) effectively improved the core features of hyperoxia-induced neonatal lung injury, alveolar simplification, pulmonary fibrosis, vascular remodeling, and vascular loss. Motor ability tests and PH studies have shown some improvement in function after early and late MEx treatments [81] (Table 1).

### 4.2. Effect of MSC-EVs on Inflammation in PH

Inflammation plays a major role in the occurrence of PH, and both innate and adaptive immune cells are related to PH [84, 85]. It destroys pulmonary vascular endothelial cells mainly through the accumulation of immune cells in pulmonary blood vessels and the release of a large number of cytokines and chemokines, which promote the proliferation of smooth muscle cells and ultimately lead to pulmonary vascular remodeling.

Immunological disorders are the basis for the development of PH. Macrophages coordinates the onset and resolution of inflammation in the lungs. Thus, manipulating lung macrophage function is a promising strategy for emerging immunomodulatory therapies including cell-based approaches [86, 87]. Under hypoxia, macrophages are activated in a lung, which induces the release of inflammatory factors, creates an inflammatory response in the lungs, and eventually causes hypoxic pulmonary hypertension. In an animal model of PH, MSC-Exos inhibited macrophage infiltration under hypoxic pulmonary hypertension, reduced the release of inflammatory factors in vivo, and reduced hypoxic/ischemic damage [88] (Table 2). MSC-Exos inhibited the influx of macrophages, proinflammatory and proliferative mediators (including monocyte chemoattractant protein-1), and hypoxia-induced mitotic factors into the hypoxic lung. MSC-Exos therapy inhibited hyperoxia-associated inflammation and changed the hyperoxic lung transcriptome. This alleviated hyperoxia-induced bronchopulmonary dysplasia, improved lung function, reduced fibrosis, and pulmonary vascular remodeling. The mechanism by which MSC-Exos works was related to regulation of the pulmonary macrophage phenotype [89] (Table 2). This effect was manifested by a low number of lung macrophages, a high proportion of alternative macrophages (M2/M1), and an increased number of peripheral blood vessels [74] (Table 2). These studies have shown that exosomes improve PH by modulating levels of immune and inflammatory factors. In fact, MSC-based therapies effectively modulate macrophages by promoting anti-inflammatory and proregressive phenotypes that correlate with histological and functional benefits in preclinical models of PH.

### 4.3. Effect of MSC-EVs on miRNAs in PH

Cells secrete miRNAs into body fluids through exosomes. Exosomal miRNAs establish connections between cells and regulate various important physiological processes by regulating the expression of target genes. More and more research is investigating the major role of exosomal miRNAs in the pathogenesis of PH [93].

MSC-EVs were divided into Exo and MV fractions and injected into MCT-treated mice. MSC-Exos, but not MV, reversed any increase in RV/LV + S. Exos derived from MCT-injured mice and patients with idiopathic PH contained elevated levels of miRs-19b, −20a, −20b, and −145, whereas miRs isolated from MSC-Exos, including miRs-34a, −122, −124, and −127, had anti-inflammatory and antiproliferative effects [77] (Table 2). Microarray analysis of miRNAs in MSC-Exos revealed 65 upregulated miRNAs, suggesting that MSC-Exos regulate pulmonary hypertension through their miRNAs. MiR-124 in MSC-Exos increased, while the downregulation of miR-124 contributed to pulmonary vascular remodeling in PH. It has been speculated that reversal of pulmonary vascular remodeling in mice treated with MSC-Exos may be due to upregulation of miR-124 expression. Intravenous injection of MSC-Exos into hypoxia-induced pulmonary hypertension mice inhibited activation of STAT3 signaling and the miR-17 family caused by hypoxia and increased expression of the miR-204 family. This broke the STAT3-miR-204-STAT3 feed-forward cycle, shifted the balance to an antiproliferative state, and improved PH, right ventricular hypertrophy, and pulmonary vascular remodeling [90] (Table 2).

By transfecting ASCs with antagomirs, low exosomal miR-191 expression inhibited HPAEC proliferation, while the agomir improved their proliferation. Similar results have been obtained in a single monocrotaline pyrrole (MCTP)-induced PH rat model after ASCs injection. MiR-191 inhibited the expression of bone morphogenesis protein receptor 2 (BMPR2) in HPAECs and PH rats. Therefore, miR-191 in ASCs and ASC-Exos plays a major role in PH by modulating BMPR2 [79] (Table 2).

Through endothelial cell (EC)-derived exosomes, miR-195 was delivered from ECs to SMCs, and that inhibited the proliferation and migration of SMCs by inhibiting the expression of serotonin transporters (5-HTT), and therefore micro-RNA may play a protective role in PH [91].

### 4.4. Effects of MSC-EVs on Genes in PH

BMPR2 is a member of the transforming growth factor *β* (TGF-*β*) receptor superfamily. It has serine and threonine protein kinase activity. The TGF-*β*/BMPR/BMP signaling pathway is active in pulmonary endothelial cells and pulmonary arteries. In patients with PH, mutations in the BMPR2 gene or abnormal signal transduction pathways have been identified, leading to abnormal expression of growth factors and inflammatory responses in vascular cells. Decreased or dysfunctional BMPR2 expression increased high mobility group AT-hook 1 (HMGA1) expression, leading to EndMT in PAEC and promoting occlusive remodeling of idiopathic pulmonary hypertension [94]. More exosomes can be isolated from growing endothelial cells of BMPR2 mutation carriers than from non-BMPR2 mutation carriers, which contain more translation-modulating tumor proteins involved in pulmonary hypertension. These transformative-regulated tumor proteins may be potential diagnostic markers of pulmonary hypertension [50] (Table 2).

MSC-Exos significantly upregulated the expression of Wnt5a in MCT-PH rats and hypoxic pulmonary vascular cells. Additionally, by silencing the Wnt5a gene, the therapeutic effect of MSC-Exos on hypoxic injury was suppressed [9] (Table 2). These illustrate that the mechanism by which MSC-Exos acts on pulmonary hypertension is related to the upregulation of Wnt5a expression.

The PAH and RV hypertrophy in hyperoxia-exposed bronchopulmonary dysplasia (BPD) mice have been corrected by MSC-CM/EXO treatment. Brain cell death was reduced and myelin sagging was reversed. Importantly, TSG-6, an immunomodulatory glycoprotein, has been detected in Exos. The knockdown of TSG-6 by NAb or by siRNA negated the obvious effect of Exos, indicating that TSG-6 is an important therapeutic molecule [10] (Table 2).

ACE2 and plasma Ang-(1–7) mRNA levels in the lung tissue of the MV group were upregulated compared with those of the PAH group. Conversely, ACE and Ang-II were decreased. However, this enhanced protection was attenuated by A-779, a Mas receptor inhibitor of the ACE2-Ang-(1–7)-Mas axis. BMMSCs-MVs changed the balance from the ACE-Ang-II-AT1R axis to the ACE2-Ang-(1–7)-Mas axis and produced positive results on MCT-induced PH in vivo, which may be a possible therapeutic mechanism for subcellular therapy with MVs [76] (Table 2).

### 4.5. MSC-Exos Improve Mitochondrial Health of PH

Although chronic hypoxia leads to glycolytic metastasis of PASMC, exosomes restore the energy balance and improve O_2_ depletion. Exosome exposure increased the expression of pyruvate dehydrogenase (PDH) and glutamate dehydrogenase 1 (GLUD1) in PASMC. Additionally, exosomes reduced the expression of sirtuin 4, an upstream inhibitor of GLUD1 and PDH, despite prolonged hypoxia. These data provide first-hand material for exosomes to improve mitochondrial function and provide new insights into the therapeutic prospects of exosomes in PAH [92] (Table 2).

In conclusion, MSC-Exos therapy opens up a promising field in the treatment of PH, proposes a new concept, and attracts increasing attention. A growing number of preclinical studies have demonstrated that cell therapy is a promising therapeutic strategy for treating refractory and uncontrollable lung diseases. The complex interplay between immune system homeostasis and mesenchymal stem cells remains not fully understood. To ensure quality control and reproducible production of functional EVs, MSC-EVs clinical translation still faces many challenges, including scalable manufacturing and identity and potency assays [95]. The Association for Clinical Research and Translation of Extracellular Vesicles discussed opportunities and challenges for developing EV-based therapeutics at preclinical and clinical levels [96]. Using MSC-EVs may have considerable advantages over using live MSCs, such as reduced side effects due to the absence of infusion toxicity [97]. Although there are still significant challenges in treating PH with stem cells-EVs, we believe these issues will be addressed in further research.

## Figures and Tables

**Table 1:** The effects of MSC-EVs against experimental PH.

References	Year	animals or cells	model	EV source	Therapeutic strategy	Impact indicators	Effects mechanisms
[73]	2021	Rats	MCT	UCMSCs-Exos	Tail vein injection once daily	Reduced RVSP and RVHI	Suppressed the pulmonary vascular remodeling and the EndMT process.

[9]	2020	Rats	MCT or hypoxia -induced cell damage model	UCMSCs-Exos	Tail vein injection once daily or added into the cells cultured medium	Attenuated RV hypertrophy and PASMC proliferation	Suppressed the pulmonary vascular remodeling and EndMT process or inhibited hypoxia-induced PAEC apoptosis

[74]	2020	Rats	Sugen/Hypoxia	MSC-EVs	Tail vein injection once daily for 3 Days	Reduced RV hypertrophy	Supressed muscularization of peripheral pulmonary vessels

[75]	2014	Rats	MCT	BMMSC-MVs	Injection for 2 weeks	Reduced RV hypertrophy and pulmonary arteriole AI and TI	Reduced pulmonary arteriole remodeling

[76]	2018	Rats	MCT	BMMSC-MVs	Tail vein injection every 2 days until 5 weeks	Relieved RV hypertrophy index, pulmonary vessel wall TI, pulmonary vessel lumen AI, the inflammation score, and the collagen fiber volume fraction	Shifted the balance from ACE-Ang-II-AT1R axis toward the ACE2-Ang-(1–7)-mas axis

[77]	2016	Mice	MCT	mMSC-Exos	Tail vein injection once daily for 3 days	Prevented any increase in RV/LV + S, pulmonary arterial WT/D	Reversed pulmonary vascular remodeling

[78]	2021	Rats	SuHx	MSC-EVs	Tail vein injection once weekly for five weeks	Reduced RVSP, RV/LV + S, and muscularization index	Reduced pulmonary vascular remodeling

[79]	2019	Rats	MCTP	ASCs-Exos	Injected for four weeks	Improved proliferation of MCTP-treated HPAECs	Regulation of BMPR2

[80]	2020	Rodents	Nitrofen administration	MSC-EVs	Intravenous infusion upon birth.	Bolstered structural aspects of the PAECM and mitigated pathological disorganization	Rapid inhibition of ECM-remodeling enzymes (LOX and MMP-9)

[81]	2020	Newborn mice	HYRX	MEx	Injected via the superficial temporal vein	Decreased pulmonary fibrosis and vascular muscularization	Ameliorated lung injury, improved alveolar simplification, pulmonary fibrosis, vascular remodeling and blood vessel loss

EVs: extracellular vesicles; Exos: exosomes; MCT: monocrotaline; MSCs: mesenchymal stem cells; UCMSCs: umbilical cord mesenchymal stem cells; BMMSCs: bone marrow mesenchymal stem cells; mMSCs: murine MSCs; ASCs: adipose mesenchymal stem cells; RVSP: the right ventricular systolic pressure; RVHI: right ventricular hypertrophy index; EndMT: endothelial-mesenchymal transition; RV: right ventricular; PASMC: pulmonary arterial smooth muscle cells; AI: area index; TI: thickness index; mMSCs: murine MSCs; RV/LV + S: right-to-left ventricle + septum wet weight ratio; WT/D: wall thickness-to-diamete; SuHx: rats treated with sugen 5416 and exposed to three weeks of hypoxia; MCTP: monocrotaline pyrrole; CDH: congenital diaphragmatic hernia; PA: pulmonary artery; ECM: the extracellular matrix; HYRX: hyperoxia; MEx: MSC-derived small extracellular vesicles.

**Table 2:** The mechanism analysis of EVs against PH.

References	Year	Disease Model	Type of EVs	Target Cell or Tissue	Molecular Mechanism	Therapeutic Effect
[88]	2018	Hypoxia	MSC-Exos	Macrophage	Inhibition of macrophage infiltration and reduced the release of inflammatory factors	Reduced hypoxic/ischemic damage

[89]	2018	BPD	MSC-Exos	Macrophage	Suppression of the proinflammatory “M1” state and augment of an anti-inflammatory “M2-like” state	Improvement of lung function, decrease in fibrosis and pulmonary vascular remodeling

[74]	2019	Sugen/hypoxia	MSC-EVs	Macrophages	Increased the ratio of alternatively to classically activated macrophages (M2/M1)	Normalized RV pressure and reduced RV hypertrophy and muscularization of peripheral pulmonary vessels

[77]	2016	MCT	MSC-Exos	Pulmonary arterial	Increased levels of anti-inflammatory, antiproliferative miRs	Prevented any increase in RV/LV; S, WT/D ratios

[90]	2012	Hypoxia	MSC-Exos	HPAECs	Increased lung levels of miR-204 and inhibited STAT3 signaling	Exerted a pleiotropic protective effect on the lung

[79]	2019	MCTP	ASCs-Exos	HPAECs	miR-191 repressed the expression of BMPR2	Inhibited HPAECs proliferation

[91]	2017	Balloon angioplasty injury on rat carotid arteries	EVs from EC-CM	SMCs	Transferred miR-195 from ECs to SMCs	Inhibited the expression of 5-HTT in SMCs and the proliferation of SMCs.

[50]	2018	Cultured BOECs	Exos from BOECs (isolated from patients harboring BMPR2 mutations)	PASMCs	Transferred TCTP from ECs to PASMCs	TCTP overexpression induced proliferation and reduced apoptosis

[9]	2020	MCT or hypoxia	MSC-Exos	HPAECs and PASMCs	Upregulated the expression of Wnt5a	The inhibition effect on EndMT and promotion adhesion ability

[10]	2018	Hyperoxia-exposed BPD	MSC-Exos	Alveolar	Detected TSG-6 in Exos	Attenuated BPD and its associated pathologies

[76]	2018	MCT	MSC-MVs	Lung tissue	Upregulated ACE2 mRNA in the lung tissues and plasma levels of Ang-(1–7)	Exerted beneficial effects against MCT-induced PAH

[92]	2019	Hypoxia	MSC-Exos	PASMCs	Increased PASMC expression of PDH and GLUD1	Improved the mitochondrial dysfunction

ASCs: adipose-derived mesenchymal stem cells; HPAECs: human pulmonary artery endothelial cells; BMPR2; bone morphogenetic protein receptor 2; MCTP: monocrotaline pyrrole; EC-CM: endothelial cells conditional medium; SMCs: smooth muscle cells; PASMC: pulmonary artery smooth muscle cells; TCTP: translationally controlled tumor protein; BOECs: blood outgrowth endothelial cells; BPD: bronchopulmonary dysplasia; TSG-6: tumor necrosis factor alpha-stimulated gene-6; NAb: neutralizing antibody; PDH: pyruvate dehydrogenase; GLUD1: glutamate dehydrogenase 1.

## Data Availability

Citations appear in the body of the article with a corresponding reference in the reference list. And the citations data used to support the findings of this study are included within the article.
